# An open source software for analysis of dynamic contrast enhanced magnetic resonance images: UMMPerfusion revisited

**DOI:** 10.1186/s12880-016-0109-0

**Published:** 2016-01-14

**Authors:** Frank G. Zöllner, Markus Daab, Steven P. Sourbron, Lothar R. Schad, Stefan O. Schoenberg, Gerald Weisser

**Affiliations:** Computer Assisted Clinical Medicine, Medical Faculty Mannheim, Heidelberg University, Theodor-Kutzer-Ufer 1-3, 68167 Mannheim, Germany; Department of Clinical Radiology and Nuclear Medicine, University Medical Center Mannheim, Heidelberg University, Mannheim, Germany; Division of Biomedical Imaging, University of Leeds, Leeds, UK

**Keywords:** Open source software, Dce-mri, Data analysis, Compartment models, Workflow

## Abstract

**Background:**

Perfusion imaging has become an important image based tool to derive the physiological information in various applications, like tumor diagnostics and therapy, stroke, (cardio-) vascular diseases, or functional assessment of organs. However, even after 20 years of intense research in this field, perfusion imaging still remains a research tool without a broad clinical usage. One problem is the lack of standardization in technical aspects which have to be considered for successful quantitative evaluation; the second problem is a lack of tools that allow a direct integration into the diagnostic workflow in radiology.

**Results:**

Five compartment models, namely, a one compartment model (1CP), a two compartment exchange (2CXM), a two compartment uptake model (2CUM), a two compartment filtration model (2FM) and eventually the extended Toft’s model (ETM) were implemented as plugin for the DICOM workstation OsiriX. Moreover, the plugin has a clean graphical user interface and provides means for quality management during the perfusion data analysis. Based on reference test data, the implementation was validated against a reference implementation. No differences were found in the calculated parameters.

**Conclusion:**

We developed open source software to analyse DCE-MRI perfusion data. The software is designed as plugin for the DICOM Workstation OsiriX. It features a clean GUI and provides a simple workflow for data analysis while it could also be seen as a toolbox providing an implementation of several recent compartment models to be applied in research tasks. Integration into the infrastructure of a radiology department is given via OsiriX. Results can be saved automatically and reports generated automatically during data analysis ensure certain quality control.

## Background

Perfusion imaging has become an important image based tool to derive the physiological information in various applications, like tumor diagnostics and therapy, stroke, (cardio-) vascular diseases, or functional assessment of organs [1, 2]. Mostly this technique is applied in magnetic resonance imaging (MRI) [3] but also emerges into the field of computed tomography (CT) [4, 5] and ultrasound (US) [6]. Especially in MRI, this technique benefits of good tissue contrast, that it is noninvasive and without the application of ionized radiation. A common approach to measure perfusion using MRI is dynamic contrast enhanced (DCE) MRI, using T1-weighted sequences to record the local signal change due to the contrast agent bolus passing through the observed area. By applying so called pharmacodynamic models to the data hemodynamic parameters like the blood flow (or perfusion), blood volume, mean transit time or the extravasation of the contrast agent from the blood stream e.g., into the interstitial space can be calculated. In recent years, added value of DCE-MR perfusion imaging has been reported in various application, e.g., for kidney [7–9], liver [10], or heart disease [11]. In prostate cancer DCE-MR perfusion imaging has developed as one part of a multi-parametric approach to stage cancer [12, 13]. It is also applied in preclinical functional imaging [14, 15]. However, even after 20 years of intense research in this field, perfusion imaging by DCE-MRI still remains a research tool without a broad clinical usage. One problem is the lack of standardization in technical aspects which have to be considered for successful quantitative evaluation, including sequence and contrast agent dose optimization [16] model selection [17], correct selection of the arterial input function [18, 19], or correction of motion artifacts [20, 21]. Recently, efforts are made to overcome this, e.g., by the Quantitative Imaging Biomarkers Alliance (QIBA) [22] of the Radiological Society of North America (RSNA) or the EIBALL – European Imaging Biomarkers Alliance [23].

The second problem is a lack of tools that allow a direct integration into the diagnostic workflow in radiology. To the best of our knowledge, apart from the work presented herein, there are only few research tools that are also integrated into a DICOM workstation [24, 25] . Research tools that allow calculations of hemodynamic parameters are developed often as offline solutions and the clinician has to transfer the large image data sets to a separate workstation for analysis [3, 24–28]. Furthermore, to include the results in the clinical workflow they have to be transferred back into the diagnostic system. However, results obtained from most research software are stored in various formats that could not easily be converted to DICOM objects to be stored in picture archive and communication systems (PACS) [24]. Certainly, the aforementioned procedure is feasible in the research context investigating small patient groups; however, in daily practice this becomes cumbersome.

Commercial software solutions to analyse DCE-MRI data exist and they allow integration into the clinical environment. This comprises products of independent companies but also solutions provided by the vendors of the MR scanners, but suffering of multi-vendor capability. Furthermore, a major problem of these solutions is that they are black-box, which means that validation and absolute benchmarking is difficult. This has real clinical implications, as demonstrated recently in the study by Heye et al. [26] which reported that a “considerable variability for DCE MR imaging pharmacokinetic parameters (*K*trans, *k*ep, *ve*, iAUGC) was found among commercially available perfusion analysis solutions” and that therefore “clinical comparability across perfusion analysis solutions is currently not warranted”.

In addition, such software is expensive in respect to the cost-benefit ratio: available commercial software solutions are often dedicated to a single application, i.e., heart, brain, or prostate perfusion [27] which does not allow for easy extension and adaption beyond the intended usage in the clinical situation [28]. Therefore, only few licenses or dedicated workstations are usually purchased which prevents ubiquitous usage [29, 30].

Recently our group presented a perfusion analysis tool (UMMPerfusion) that aimed at overcoming some of the aforementioned problems [31]. In the initial version of our software, we provided means for quality assurance by visualizing the arterial input function (AIF) online while drawing its respective region of interest and by generating automatically a report logging all settings of the respective data analysis session. The software itself was designed as a plugin for the Open Source DICOM Workstation OsiriX [32, 33] which can be fully embedded into the radiological workflow [30] and thereby, calculated results by our software, too. The decision to select OsiriX, besides that it has been installed for research and clinincal use in our Radiology department was that OsiriX became a very popular and powerful software with more than 40.000 users worldwide at very low costs. By implementing the perfusion anaylsis software as OsiriX plugin and Open Source, we hope to reach a large number of users and to bring perfusion imaging forward by providing analysis software.

To calculate hemodynamic parameters, however, so far only a model-free deconvolution approach was implemented. Pharmacokinetic models reported in the literature [34] offer additional parameters, e.g., permeability or extravascular extracellular volume and describe the tissue in more detail.

In this paper, we will present recent extensions of our software. This comprises the implementation of several well established compartment models and their integration into the plugin and the quality management developed for this software.

## Implementation

### Perfusion models

Besides the existing deconvolution approach described in detail in [31], five additional models were implemented, namely, a one compartment model (1CP), a two compartment exchange (2CXM), a two compartment uptake model (2CUM), a two compartment filtration model (2FM) and eventually the extended Toft’s model (ETM). A detailed description with theoretical background [35] and the reference implementation in IDL (Exelis VIS, Boulder, CO) of the single models is detailed in [36]. The implementation of the compartment models in this work was translated from IDL to C/C++. Table 1 shows the different parameters to be obtained by the implemented models.

**Table 1:** Compartment models and their pharmacokinetic parameters and respective units as implemented in our software. Please note that Ktrans = E*Fp and, for the ETM, by definition PS = Ktrans

	Plasma Flow (Fp) (ml/min/100 ml)	Plasma MTT (s)	Plasma Volume (ml/100 ml)	Interstitial MTT (s)	Interstitial Volume (ml/100 ml)	Extraction Fraction (E) (%)	Permeability-surface area product (PS) (ml/min/100 ml)	Ktrans (ml/min/100 ml)
1CP	x	x	x					
2CUM	x	x	x			x	x	x
2CFM	x	x	x	x		x	x	x
2CXM	x	x	x	x	x	x	x	x
ETM			x	x	x		x	x

We have chosen to model the capillary bed in terms of arterial plasma concentration (c_a_), tissue plasma concentration (c_p_), plasma volume (v_p_) and plasma flow(F_p_) as in [37]. Others use arterial blood concentration (c_a,b_), tissue blood concentration (c_b_), blood volume (v_b_) and blood flow (F), and use the term ‘AIF’ for c_a,b_. There are no experimental indications that either plasma- or blood parameters form better biomarkers, so the choice between the alternatives is a matter of historical convention. All equations can be translated between conventions by inserting the haematocrit of arterial blood (Hct_a_), and the haematocrit of tissue blood (Hct_t_). In practice, the blood concentration c_a,b_ is measured from an ROI in a feeding artery. c_a_ is then derived by dividing _ca,b_ by 1-(Hct_a_) and a known value for the arterial haematocrit Hct_a_. A measured value for the individual subject should be used when available; if not, a standard value such as Hct_a_ = 0.45 is often used [38].

All models are fitted to the measured data by non-linear least square optimization, e.g., by the Levenberg-Marquardt-Algorithm (LMA). In our implementation, we incorporated the LMA implementation by Markwardt et al. (mpfit, version 1.2) [39]. The mpfit algorithm thereby serves as a general solver of the optimization problem. The respective compartment model was implemented as a function that is passed to the solver. Thereby, a modularization and easy extension of our plugin is possible. To add a further model, only the model function has to be implemented.

Another benefit of this modularization is that parameters related solely to the fit algorithm (stopping criteria, number of iterations, etc.) can be configured and maintained globally and be provided to all models. This prevents different settings for each perfusion model and allows comparison of results across perfusion models. In the current implementation, all such parameters have been adopted from the reference implementation in PMI [40]. Each parameter can be altered by the user according to their needs. Also, to constrain the fitting algorithm, the initial parameters of the compartment models can be fixed or upper and lower bounds can be set. All user defined settings can be saved as preferences for future analyses. Furthermore, the preferences can be exported to be distributed within or across institutions or to have common preferences for e.g., a certain study or application. It is saved in the property list format (plist) provided by the Mac OSX operating system [41].

### Data analysis options

In our software, for the newly implemented compartment models, two ways to analyze the data were implemented; based on a region of interest (ROI) and calculation of parametric maps. Furthermore, the user can choose to compare different ROIs (within the same data set) using one model or to compare the different compartment models among each other.

#### ROI based analysis

The ROI approach allows selecting a specific area within the data set to be analyzed. Within this (tissue) ROI, all time intensity curves of the enclosed pixels are averaged and then the respective model is fitted to this curve.

#### Calculation of parametric maps

In the map mode, a voxel wise fitting of the compartment model is performed. Although means for parallelization of the calculations are implemented within our software using Grand Central Dispatch [42], this approach can be time consuming, especially in large datasets and within the background voxels where no meaningful fit results are expected and the fit algorithm will probably not converge. Therefore, we force the user to select a rectangular region around the respective part in the image to be analyzed. This ROI is propagated throughout the whole 3D stack and within this volume, voxel wise fitting is performed.

#### Comparing multiple perfusion models

Choosing the right perfusion model to analyze the obtained DCE-MRI data is critical [3, 43]. As outlined before, we implemented a range of models with different amount of free parameters to be fitted. For example, the 1CP has two free parameters while the 2CXM has four, i.e., they model the tissue physiology differently.

In general, a too simple model might not capture the physiology within the tissue while a too complex model might overfit or the large amount of fitted parameters cannot be estimated under stable conditions. Therefore, comparing different models on the data at hand and estimating the goodness of fit is a strategy to select the most appropriate model.

To make this most feasible and easy, in our implementation, the user can switch between the different perfusion models while our software keeps recent settings like signal normalization, baseline, the selected AIF and tissue ROI and therefore, provides them directly to the selected model. Thereby, common errors during perfusion analysis like nonsimilar ROI selection, changes in prerequisites are avoided. The user just simply needs to press the ‘Generate’ button for computing the additional model with all previous settings.

To automate this comparison, we added functionality to loop over the different compartment models and calculate for each the respective pharmacodynamic parameters. It is implemented for ROI based data analysis and the calculation of parametric maps. To support the user to evaluate the results two goodness-of-fit (GOF) measures are provided with the results, namely the *χ*^2^ error and the Akaike information criterion (AIC) [44]. In case of the calculations of parametric maps, respective maps of *χ*^2^ error and AIC are provided. When comparing several models, given the reported AIC values of each model, the relative information loss, i.e., how good the model fit the data, can be calculated

$$ {e}^{\frac{AI{C}_i- AI{C}_{\min }}{2}} $$

where AIC_i_ is the actual AIC of the model I and AIC_min_, the minimum AIC value of all models.

#### Comparing several ROIs with one model

Another way to analyze DCE-MRI data is to compare different (tissue) ROIs within the data set, e.g., to see differences between healthy and diseased tissue or between paired organs like the kidney. To foster automation here too, we also implemented an option to loop over all (tissue) ROIs within the data set and calculate the selected model for these.

### Software design

**Fig. 1:**
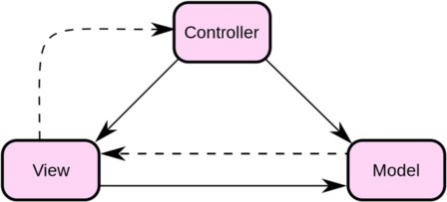
Sch. of the model-view-controller software design pattern. Arrows depict interactions between the three components. Source: https://de.wikipedia.org/wiki/Datei:ModelViewControllerDiagram2.svg#filelinks

The design of our software follows the Model–View–Controller (MVC) design pattern [45, 46]. Thereby, the communication between the user, the graphical user interface (GUI) for visualization (view) and the model is handled via a so called controller (cf. Fig. 1). This allows a separation between the computation and the visualization/ user interaction in our system and makes our plugin modular and extensible. For example, the design of the GUI can be changed without the need to change the model (e.g., computation of the compartment models). The controller steers the communication between view and model. In the following two examples depicting briefly the software design are presented.

#### Graphical user interface

In our implementation the GUI represents the Controller of the MVC concept. It allows the user to steer the calculation implemented in the model. Results of the calculation are passed on to the View by calling the respective visualization function provided by OsiriX (so called 2D viewers).

In the initial version of the plugin, only the fast model-free deconvolution algorithm was implemented [28]. Since, we implemented several other models the GUI was extended. Thereby, care was taken to not overload the interface so that the user can easily work with the software.

**Fig. 2:**
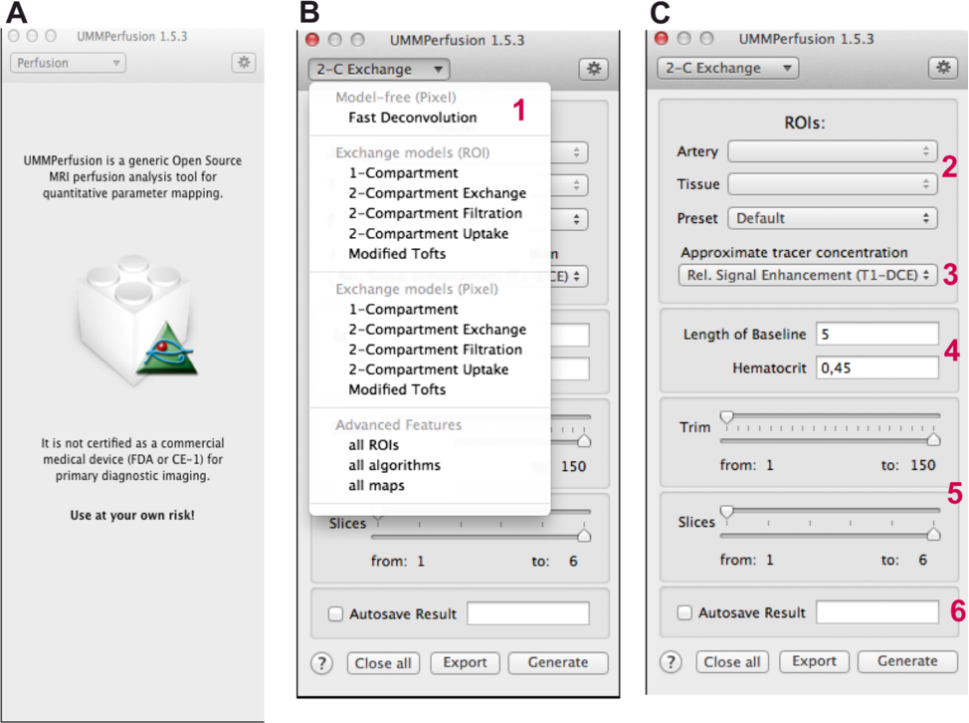
Graphical User Interface of the UMMperfusion software. **a** initial view after loading the plugin, **b** drop down box showing the different models and analysis modes, **c** example of a specific panel (here 2CXM) to perform the calculation. The red numbers (from 1 to 6) depict the workflow for analyzing DCE-MRI data using our software

At start the user is presented just a top down menu to select their perfusion model of choice (see Fig. 2a). For each perfusion model a panel showing the inputs is visualized when selected from the top down menu (cf. Fig. 2b). We designed the GUI in such way that workflow (selection of input parameters) needed to calculated the model was mostly kept the same. Overall, up to six steps (see Fig. 2, red numbers) have to be performed to obtain a result. This comprises to select the model of choice (1), to select the arterial input function (AIF) and a region of interest (ROI) of the respective tissue to be analysed (2), selection of the type signal normalization (3) and the baseline (4), i.e., number of time points to include for the signal normalization. Moreover, the user has the option to select a range of slices from the 3D volume and to trim the time series (5). Eventually, the user can set a name prefix (6) which is added to the results. This might be beneficial to tag results if different parametrization of a model during a data analysis session is performed. Ticking the ‘autosave’ checkbox allows for saving the results directly to the OsiriX database automatically. Saving results can be also triggered manually by hitting the export button. After successfully following the above steps, the ‘Generate’ button starts the calculations.

**Fig. 3:**
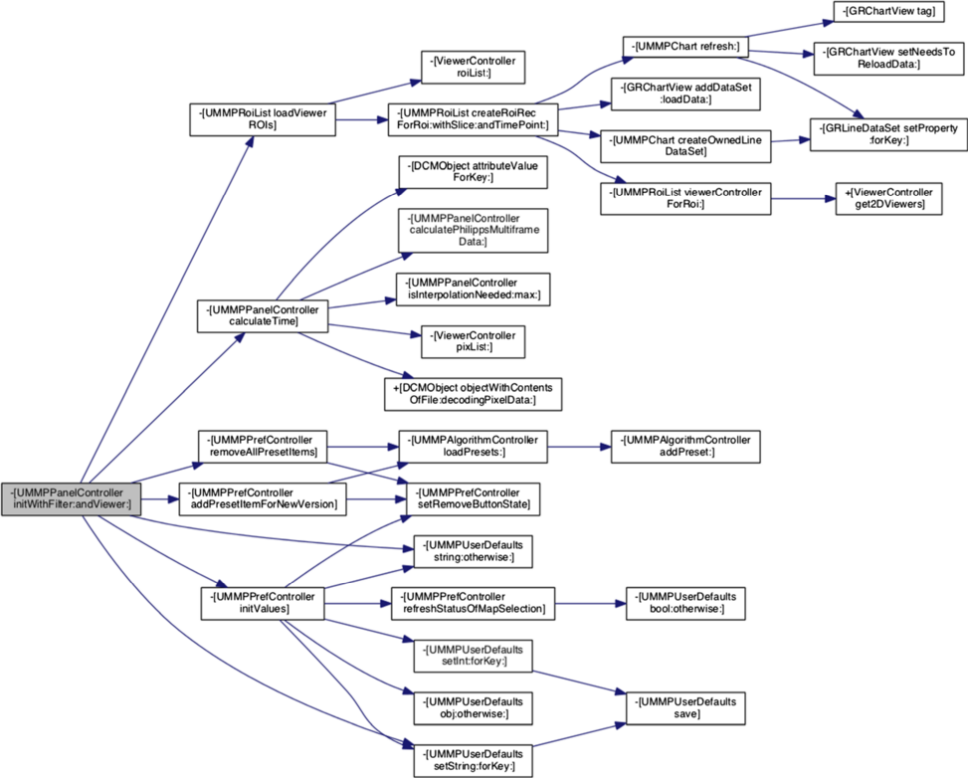
Call graph of the UMMPPanelController class. This controller steers the visualization of the individual panels (see Fig. 2c) of the respective models. It also calls several other objects (e.g., the algorithm controller for calculating the compartment models or classes for visualizing the AIF) within the plugin

Technically, after initialization of the plugin, the GUI depicted in Fig. 2 is created by a controller to steer this panel following the above described MVC design pattern. Figure 3 shows, as an example, the call graph of the initialization method depicting the connection to Views, to data management objects (e.g., to store information about ROIs), and to controller that supervises the actual computation.

#### Compartment models

To realize a flexible solution and to easily extend our software by possible other compartment models, we followed the object-oriented programming paradigm.

**Fig. 4:**
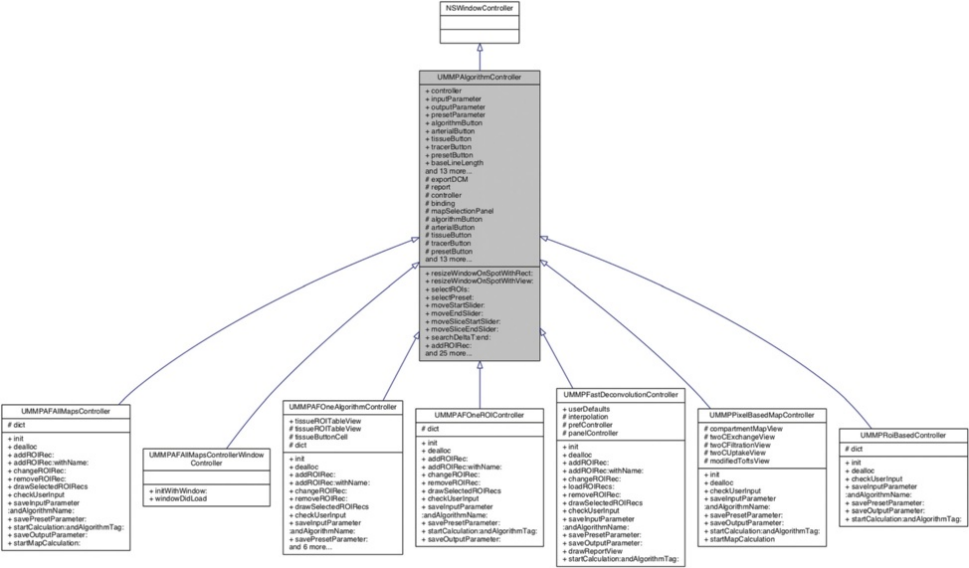
Class diagram of UMMPAlgorithmController. It depicts the respective classes and their methods. The arrows show the inheritance of the subclasses. Here, the subclasses implement the different data analysis option (ROI-based, parametric maps, the deconvolution approach, and the advanced options to compare models)

Figure 4 shows the inheritance diagram of the UMMPAlgorithmController that provides a general class for calculating a perfusion model based on the GUI inputs and the DCE-MRI data. From this general class, it could be regarded as a template, specific sub controllers are inherited. These hosts the actual implementation needed to e.g., calculate a compartment model and provide parametric maps as a result. Thereby, on the level of this controller, only an interface to the actual compartment model is implemented. The implementation of the respective model is encapsulated and called as a method by the instanced controller object during run time.

To implement a new compartment model, only three steps have to be performed: a) the respective implementation of the model, b) extension of the sub controller to interface the model, and eventually to adapt the GUI appropiatly to be able to select the model.

### Quality management

Besides the technical (implementation of the algorithms) and medical/physiological (modeling the perfusion in tissue) aspects of this software, another part of this software is quality management. On the one hand, this comprises testing and evaluation but also documentation of the software to allow for certification of the software for clinical use. The UMMPerfusion plugin was certified in-house according to the German Medical Product law. Besides the necessary documents, e.g., risk analysis etc., we designed a testing scheme and corresponding reference datasets [28] to continually evaluate changes to the software. In addition, we also provide a bug tracking system so that user can report errors or problems with the software but also suggestions of new features. All of this is provided via an online platform called OpossUMM (http://www.opossumm.de) which is freely accessible.

Moreover, we also implemented means to support the user in its daily work to detect errors arising from the data analysis. This comprises a preview of the AIF and a report automatically generated and saved with the patient record. The AIF preview display thereby is update immediately if the user alters the corresponding ROI, e.g., by resizing or moving it. Thereby, the user can, before performing any calculations, check if a correct AIF was selected and prevent results hampered by this. The report gives an overview of all user selected parameters (AIF, baseline, etc.) of the respective data analysis. Furthermore, it also visualizes the selected ROIs but also the initial parameters of the fitting algorithm. By this, a documentation of the steps taken to perform a data analysis is given allowing for a retrospective quality check, also.

## Results and discussion

Our software plugin was implemented and tested on Mac OSX systems version 10.8.x using OsiriX versions 5.5.1, 5.6, and 5.9. Apart from these OsiriX versions, the software may work but no tests by the authors were performed so far.

**Fig. 5:**
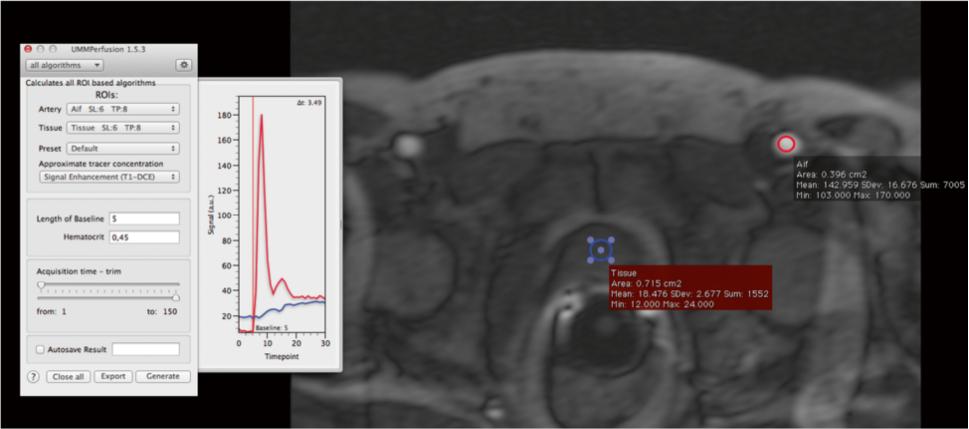
Display of the GUI and the AIF (*red curve*) for example DCE MRI data set of the prostate. Here, the AIF was selected in one of the Iliac artery. The *blue curve* is the mean signal intensity curve of prostate tissue corresponding to the blue ROI

**Fig. 6:**
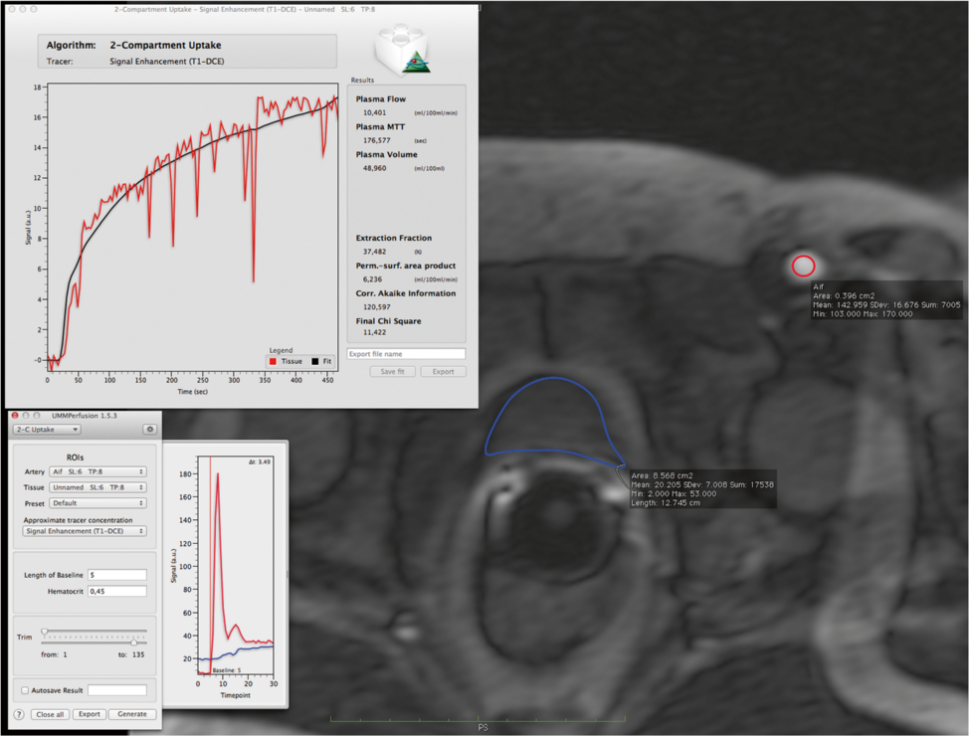
Example of ROI-based data analysis and result for a DCE-MRI of the prostate. The window in the top left area of the figure depicts the result of this analysis, showing the fit (*black curve*) to the data (*red curve*) and also listing the calculated parameters and GOF measures

**Fig. 7:**
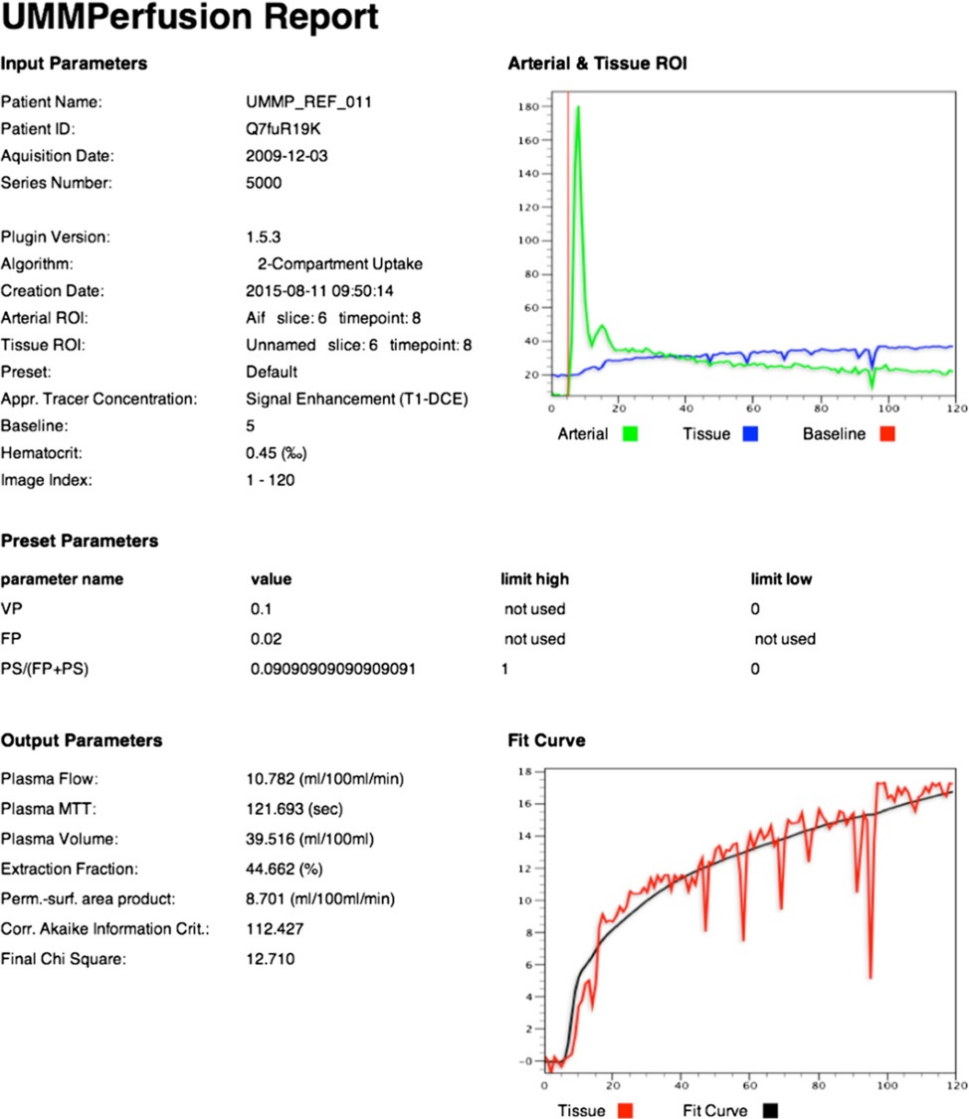
Report created from the data analysis depicted in Fig. 6. This report is stored as DICOM object in the OsiriX database together with the patient record. Besides the actual results, this report lists the employed model, all relevant parameters set during the analysis (e.g., selection of baseline), and also the initial parameters and limitations passed to the fit algorithm. Corr. Akaike Information Crit. and Final Chi Square depict measures of the goodness of the fit

Figure 5 depicts the AIF display and the GUI for an example data analysis of the prostate. The result of this ROI based analysis using the 2CUM is depicted in Fig. 6 while Fig. 7 shows the corresponding report that is created when exporting the data into the OsiriX database. The report is a DICOM object that can be archived with the patient data into PACS systems.

In the ROI based analysis as well as for the report, the calculated parameters derived from fitting the model to the data are listed. Also, the fit itself to the data is visualized in a plot. To assess the quality of the fit not only visually, we provide two goodness-of-fit measures (*χ*^2^, AIC). While the *χ*^2^ can be used to judge if the model fit was good, the AIC can be used to compare two or more models given the data.

**Fig. 8:**
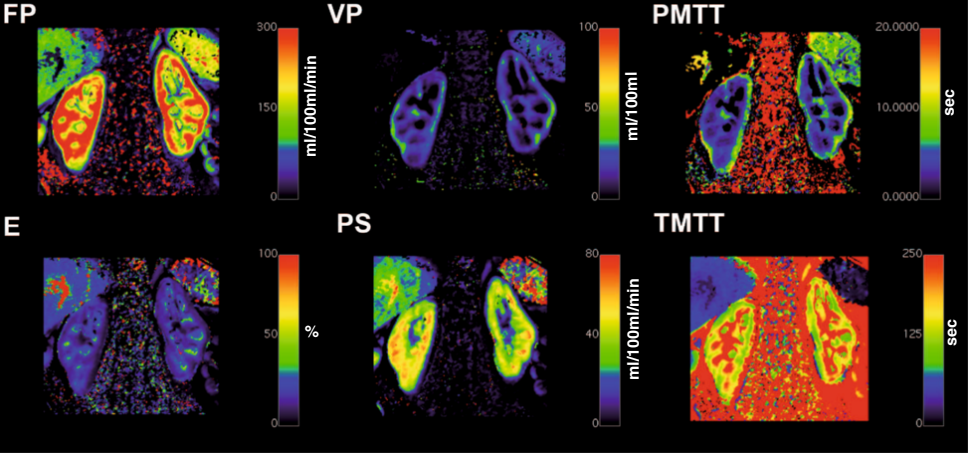
Parametric maps generated using UMMPerfusion and the 2CFM. *Top row* from *left to right*: plasma flow (FP), plasma volume (VP), plasma mean transit time (PMTT). *Lower row* from *left to right*: extraction fraction (E), permeable surface area product (PS), and tubular mean transit time (TMMT)

Similar, the results of the other implemented compartment models for a ROI based analysis would look alike. Analyzing the data using the pixel based calculation will result in a parametric map for each of the respective parameters of corresponding compartment model. As an example, Fig. 8 depicts such an analysis for a DCE-MRI data set of the kidney employing the 2CFM.

To evaluate the implemented compartment models we used a procedure previously described in [31] utilizing a reference test data set constructed from a DCE-MRI data set of the prostate. A time series with 100 time points was constructed taking a matrix of 8 × 4 pixels as one slice. Half of these pixels were taken from a vessel representing the AIF in the original data while the remaining pixels were sampled from prostate tissue. To calculate reference values for each compartment model, the software PMI [40], an authorative research tool for perfusion analysis was selected. Obtained reference values for each parameter and the corresponding values of our software were compared. In all settings, no differences between reference and our implementation were detected (see Table 2). By this we conclude that the implementation of the algorithm is technically correct.

**Table 2:** Resulting pharmacokinetic parameters calculated by the different models and the reference test data set. Model evaluation was performed by the ROI-based approach, i.e., selecting the AIF as ROI in the upper row of the test data set and the tissue ROI in the lower row (see [31] for details on the test data set). For our test data set we do not expect physiological reasonable values but identical results to verify the technical correct implementation of the algorithms in UMMPerfusion

Parameter	Reference	UMMPerfusion
1 CP		
Plasma Flow	0,148	0,148
Plasma MTT	0,012	0,012
Plasma Volume	0,033	0,033
2 CXM		
Plasma Flow	0,056	0,056
Plasma MTT	0,092	0,092
Plasma Volume	0,133	0,133
Interstitial MTT	73684,672	73684,672
Interstitial Volume	0,129	0,129
Extraction Fraction	0,149	0,149
Perm.-surf. Area product	0,088	0,088
2 CFM		
Plasma Flow	0,264	0,076
Plasma MTT	0,03	0,03
Plasma Volume	0,076	0,076
Tubular MTT	767313,375	767313,375
Tubular Flow	0,166	0,166
Extraction Fraction	0,001	0,001
2 CUM		
Plasma Flow	0,032	0,032
Plasma MTT	0,011	0,011
Plasma Volume	0,033	0,033
Perm.-surf. Area product	0	0
Extraction Fraction	0	0
ETM		
Plasma Volume	0,095	0,095
Ktrans	0,122	0,122
Interstitial MTT	0,023	0,023
Interstitial Volume	0,007	0,007

In order to process data by our plugin, two prerequisites are required, a) the data has to be in DICOM format and b) it must be loaded into the OsiriX 4D viewer. To further evaluate the robustness of our software, also for processing image data from different vendors, perfusion data sets from the three main vendors of clinical MR systems (Siemens, GE, Philips) were collected and processed successfully. The major challenge in processing data sets of the different vendors is that information of the temporal resolution is stored differently in the DICOM headers, especially for the Philips multi frame storage format (see Table 3). If no such timing information can be extracted from the DICOM header, at the moment, no calculation of the models is possible. We are currently also testing to read DICOM data provided by small animal scanners (Bruker). DCE-MRI data of 2D acquisitions could be successfully analysed.

**Table 3:** DICOM header tags addresses in hexadecimal notation used by UMMperfusion to derive the temporal resolution of the DCE-MRI series. For the Bruker DICOM, at the moment only 2D + t data can be processed, the time information is calculated from TR and the number of acquired images

Vendor	DICOM tags used for reading temporal resolution
Siemens	“0X0008,0X0032”
GE, Philips single frame	“0X0018,0X1060”
Philips multiframe	“0X0008,0X0033”
	“0X0008,0X0032”
Bruker	“0X0018,0X1310”
	“0X0018,0X0080”

**Fig. 9:**
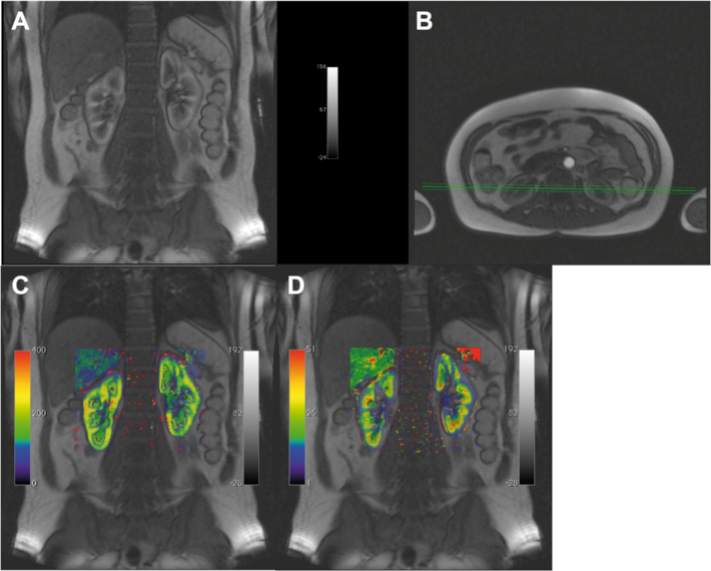
Example using a data set with different slice orientation and UMMperfusion. **a** and **b** two slices from the DCE-MRI acquired following the protocol described in [57], comprising four coronal slices (**a**) and one transversal slice (**b**). **c** map of the plasma flow calculated by UMMPerfusion and the 2CFM superimposed on (**a**, **d**) map of the extraction fraction derived superimposed on (**a**), respectively

In addition special slice positioning (e.g., 1 transversal slice, 4 coronal slices) reported in the literature [1, 7] caused problems when loaded into the OsiriX 4D viewer, a prerequisite for our plugin to detect the temporal domain of the data. Using an option in OsiriX to resort this data, the 4D viewer could be opened and thereafter, our plugin could process this data without problems (see Fig. 9).

A current drawback of our implementation is that the non-linear fitting of parametric maps is time consuming. To reduce the computation time, at present a rectangular ROI has to be placed in the DCE-MRI image series. To further improve the computational speed of the calculating parametric maps, a linear least squares approach as proposed by Flouri et al. [36] will be explored. Furthermore, an implementation of the compartment models in a Graphics Processing Unit (GPU) will be considered.

Besides our plugin several research tools exist for perfusion analysis in DCE-MRI [47–52] which might allow for benchmarking and comparison of the different solutions. In this work, we only compared our algorithms against the reference implementation in PMI [40] to verify the technical correctness. A comparison of PMI against other perfusion analysis software using the QIBA protocol and different levels of noise was reported by Cron et al. [53]. Beuzit et al. compared our plugin to four other software solutions, including commercial software from all three main vendors using simulated and measured data [54]. In this study, the ETM was used and the authors reported a bias for all solutions and pharmacokinetic parameters ranging from 0.19 min^−1^ to 0.09 min^−1^ for Ktrans, −0.15 to 0.01 for ve, and −0.65 to 1.66 mmol/L^−1^/min. In both studies the variances in the parameters between the different software solutions were attributed to various reasons. Cron et al. observed increased unphysiological values with increasing noise while Beuzit et al. stated that probably the (not documented) fitting routine might have had an influence on the results. As stated by Heye et al. such comparison might be in general difficult [26], especially for the commercial solution since little is known about their implementation. Available research tools are implemented on various platforms, requiring different input formats and outputs and eventually implemented different fitting algorithms which make a comparison difficult to interpret. Furthermore, data sets with known true values or available gold standard and fully control on the measured or simulated data should be employed when comparing and validating software to minimize e.g., inter patient variability [55]. All this implies that there is a need in standardizing the DCE-MRI perfusion analysis.

Compared to the above mentioned research tools our plugin underwent an in house certification process. This process which involves risk analysis and thorough documentation also gave insights howto improve the workflow, the structure of the source code and to prevent errors caused accidently by users and thereby improve stability of the plugin. Eventually, it allows for using our plugin for research but also for clinical routine [56]. All documents and procedures of this certification are documented at our website (http://www.opossumm.de) to help others to perform an in house certification by themselves or to adapt the procedure according to their local regulations.

## Conclusions

We developed open source software to analyse DCE-MRI perfusion data. The software is designed as plugin for the DICOM Workstation OsiriX. It features a clean GUI and provides a simple workflow for data analysis while it could also be seen as a toolbox providing an implementation of several recent compartment models, adapted from the software PMI, to be applied in research tasks. Integration into the infrastructure of a radiology department is given via OsiriX. Results can be saved automatically and reports generated automatically during data analysis to ensure certain quality control.

## Availability and requirements

Compiled binaries and source code of our Open Source software is available for download via the OpossUMM platform (http://www.opossumm.de). The software requires at least Mac OSX 10.8.x and was tested with OsiriX versions 5.5, 5.6 and 5.9. For optimal performance and to handle large datasets, the installation of the commercial available 64bit extension of OsiriX (now included in OsiriX MD) is suggested.

### Ethics approval

The data were acquired in different studies at our institution and were retrospectively selected for this paper. No permission to use the data in this study was required, however, for the data acquisition and retrospective further usage local IRB approval (Institutional Review Board II, Medical Faculty Mannheim, Heidelberg University) and written consent of the patients/ volunteers invoveld was obtained at time of the original study. All patient identifing information has been removed prior to the data analysis.

## Abbreviations

MRI: magnetic resonance imaging
DCE: dynamic contrast enhanced
AIF: arterial input function
MVC: model-view-controller
ROI: region of interest
1CP: one compartment model
2CXM: two compartment exchange model
2CUM: two compartment uptake model
2CFM: two compartment filtration model
ETM: extended Toft’s model
LMA: levenberg-marquardt-algorithm
GUI: graphical user interface
GOF: goodness-of-fit
TR: repetition time
